# Supplementary material for: Improving food security of farming households in Nigeria: Does broiler outgrowers’ program make any difference?
Source: PLoS One. 2023 Sep 21;18(9):e0291611. doi: 10.1371/journal.pone.0291611 (PMC10513292; doi:10.1371/journal.pone.0291611)
Supplement: S1 Appendix — (DOCX) [file pone.0291611.s001.docx]

# Supporting information

# APPENDIX

**OBAFEMI AWOLOWO UNIVERSITY, ILE-IFE, OSUN STATE**

**IMPACT OF OUTGROWERS PROGRAMME ON FOOD SECURITY STATUS OF SMALLHOLDER POULTRY FARMING HOUSEHOLDS IN OSUN STATE, NIGERIA.**

This questionnaire is designed for academic purpose, to obtain information on the impact of outgrowers’ programme on food security status of smallholder poultry farming households in southwest Nigeria. To this end, respondents are kindly implored to please patiently fill the questionnaire with sincerity. This is a purely academic exercise and any information provided will be treated with absolute confidentiality.

**SECTION A: SOCIO-ECONOMIC CHARACTERISTICS**

1. Local Government Area …………………………………………………………

2. Town/Settlement …………………………………………………………………

3. Age of respondent ………………………………………………

4. Gender A. Male [ ] B. Female [ ]

5. Marital status A. Single [ ] B. Married [ ] C. Divorced/separated [ ] D. Widowed [ ]

6. What is your educational qualification?

A. No formal education [ ] B. Quranic School [ ]

C. Did not complete Primary School [ ] D. Attempted secondary school [ ]

E. Completed secondary school [ ] F. Attempted tertiary education [ ]

G. Completed tertiary education [ ]

7. How many family members do you have? ……..……….. No. of spouse ………………. Children ……………..

8. How many people live with you and depend on you for survival? …………………….……..

9. Please, kindly fill the table below about the age of your children

| **Age** | **Male Child** | **Female Child** |
| --- | --- | --- |
| **Below 15 years** |  |  |
| **Between 15-35 years** |  |  |
| **Above 35 years** |  |  |

10. How many of your children between 15-35 years old work in your poultry farm? …………………..

11. Do you belong to poultry farmers’ association? …………………………………………………….

12. If yes, are you an officer of the association? A. Yes [ ] B. No [ ]

13. What post do you hold in the association? ………………………………………….

a) What are the benefits you get for belonging to the poultry farmers’ association?

1. …………………………………….
2. ……………………………………

b) If you are not a member of poultry farmers’ association, why?

1. …………………………………….
2. …………………………………….

14. Do you belong to any other association(s)? Yes [ ] No [ ]

15. If yes, what type of association? (a) Poultry Farmers’ Association (b) Cooperative Society (c) Others (Specify) …………………………………………………

16. How many years have you been producing poultry birds? ………………………………………

17. What is the source of fund to start your poultry farm? A. Personal Saving [ ] B. Friends and Relatives [ ] C. Commercial Banks [ ] D. Government Agencies [ ] E. Money Lenders [ ] F. NGOs [ ] G. Others (specify)……………….

18. How much did you use to start your poultry farm? ……………………………………………………..

19. Do you have access to credit facility for your poultry farm? A. Yes [ ] B. No [ ]

20. (A) Do you have access to extension agent’s visit? A. Yes [ ] B. No [ ]

(B) If yes, how often? A. Rarely [ ] B. Once in a while [ ] C. Regularly [ ] D. Very Often [ ] E. Always [ ]

(C) What are the major benefits you gained from extension visits to your farm?

1. ………………………………………….
2. ………………………………………….

21. (A) Have you received any form of training on poultry production? A. Yes [ ] B. No [ ]

(B) If yes, list the training(s)

1. …………………………………………
2. …………………………………………

(C) How frequent? Weekly [ ] Fortnightly [ ] Monthly [ ]

(D) How has the training helped improve your enterprise?

1. …………………………………………....
2. .…………………...…………………........
3. …………………………………………....
4. ………………………………………........

22. (A) Do you receive training from other source aside outgrowers programme? Yes [ ] No [ ]

(B) If yes, list the training(s)

1. ………………………………………………...
2. ………………………………………………...
3. ………………………………………………...
4. …………………………………….….............

23. How do you sell your products? A. Wholesaling [ ] B. Retailing [ ] C. Both [ ]

(A) If retailing, where do you sell? (**circle as appropriate**) A. Open market [ ] B. Farm gate [ ]

C. Supermarket [ ] D. Eateries and Restaurants [ ] E. Individual contact [ ]

F. Others (specify) ………………………………..

(B) If wholesaling, who are your buyers? ) A. Supermarket [ ] B. Processors [ ]

C. Retailers [ ] D. Eateries and Restaurants [ ] E. Government agencies [ ]

F. Others (specify) ………………………………..

24. How many of the people living with you are working outside your farm? ………………………………….

25. Do you have any other sources of income outside poultry farm? A. Yes [ ] B. No [ ]

26. If yes, complete the table below;

| **Off-farm Occupation** | **Annual Income Generated (₦)** |
| --- | --- |
| **i)** |  |
| **ii)** |  |

27. Please indicate your non-poultry source(s) of income (you are allowed to tick more than one)

A. Work in your own activity and you are own boss (Self-employment) [ ]

B. Work for government civil service (Civil servant) [ ]

C. Work for a private firm (Private worker) [ ]

D. Receive money from migrant family members (Remittances) [ ]

E. Pension, social allowances (Pensioner) [ ]

28. Have you heard about outgrowers programme? A. Yes [ ] B. No [ ]

29. If yes, how did you hear about NIRSAL outgrowers programme?

……….....……………………………………………………………………………….

30. Why are you not participating in the programme? ………………………………………………………………………….…………………………….…………………………………………………………………………………

**SECTION B: FARM INCOME OF POULTRY OPERATION**

31. Farm Input Costs and Output Price

How much did it cost you to purchase and sell these birds?

*Fill the details for the poultry farm inputs and outputs provided below*

| **Bird type** | **Poultry production cycles per year** | **Flock size (Quantity)** | **Purchase price /unit** | **Number sold** | **Selling price /kg (Naira)** | **Average weight at sales (kg)** | **Stocking density (number/cage)** | **Average age at sales (weeks)** |
| --- | --- | --- | --- | --- | --- | --- | --- | --- |
|  |  |  |  |  |  |  |  |  |

**SECTION C: PARTICIPATION IN OUTGROWERS’ PROGRAMME**

32. How did you get to know about the outgrowers’ programme? ………………………………………………………………………

33. What year did you start participating in outgrowers’ programme? ………………………………………………………………..

34. What year did you start to benefit from outgrowers’ programme? ………………………………………………………………...

35. Are you still a beneficiary of outgrowers’ programme? Yes [ ] No [ ]

36. Are you a beneficiary of other government intervention(s) aside outgrowers’ programme? Yes [ ] No [ ]

37. If yes, name the programme(s) scheme(s)

1. ………………………………………….
2. ……………………………………………

38. What are the conditions you must meet to participate in outgrowers’ programme?

1. ……………..………………………………..
2. …….………………………………………..
3. ………..…………………………..………..
4. ……………………………………..………..

39. Do you participate in the outgrowers’ programme in the last one year?

A. Yes [ ] B. No [ ]

40. If yes to question 39, how many production cycles have you participated in the outgrowers’ programme?

……………………………………

41. If no to question 39, why did you stop participating in the programme?

1. ……………………………………………….
2. ………………………………………………
3. ……………………………………………....

42. How will you rate your experience with outgrowers’ programme?

Excellent [ ] Good [ ] Fair [ ] Poor [ ]

43. (A) Do you find it easy accessing support from outgrowers’ programme? Yes [ ] No [ ]

(B) If no, why?

1. ………………………………………………
2. ………………………………………............

44. Who are the off-takers of your poultry outputs ……………………………………………………………………………………………

45. Do you sell poultry outputs directly to the public? A. Yes [ ] B. No [ ]

46. If yes, what proportion of your products do you sell to public in percentage? ………………………………………………

47. *In table below, provide information on average amount of poultry output (kg) sold to the off-takers*

| **Bird type** | **No. of production cycles** | **Average number sold** | **Selling price /kg** | **Average weight at sales (kg)** | **Average Age at sales (weeks)** |
| --- | --- | --- | --- | --- | --- |
| Broiler |  |  |  |  |  |

48. Please, provide details of the inputs received from anchor(off-taker) in the last one year

| **Farm Input** | **Off-taker’s Name** | **Quantity** | **Cost /unit** | **How many times per year?** |
| --- | --- | --- | --- | --- |
| Feed (kg) |  |  |  |  |
| Broiler chicks |  |  |  |  |
| Layer chicks |  |  |  |  |
| Fumigants |  |  |  |  |
| Drugs/vaccines |  |  |  |  |

49. What is the prevailing interest rate on outgrowers’ credit? ………………………………………………………………………

50. What proportion of the poultry output did you agree to payback as in-kind loan to the off taker? …………………...

**SECTION D: Food Security**

56. Qualitative questions on household food security

| S/N | Question | Never | Occasional | Regularly | Very often |
| --- | --- | --- | --- | --- | --- |
| 1 | Did you worry that your household would not have enough food in the past four weeks? |  |  |  |  |
| 2 | Did you or any household member not able to eat kinds of food you preferred because of lack of resources in the past four weeks? |  |  |  |  |
| 3 | Did you or other household members ever skip a meal because there was not enough money to buy food in the past four weeks? |  |  |  |  |
| 4 | Did you or other household member have to eat some foods that you really did not want to eat because of lack of resources to obtain other type of food in the last four weeks? |  |  |  |  |
| 5 | Did you or any household have to eat any smaller meal than you felt you needed because there was not enough to eat in the past four weeks? |  |  |  |  |
| 6 | Did you or any household member have to rely on only a few kinds of low-cost food to feed your children because you were running out of money to buy in the past four weeks? |  |  |  |  |
| 7 | Did you or other household member have to eat fewer meals in a day because there was no food to eat in the past four weeks |  |  |  |  |
| 8 | Did you or other household member have to go a whole day and night without eating anything because there was not enough food in the past four weeks? |  |  |  |  |

57. Questions on coping mechanism used by the household

| S/N | Coping mechanism | Never | Occasional | Regularly | Very often |
| --- | --- | --- | --- | --- | --- |
| 1 | Borrowed money to buy food or got food on credit |  |  |  |  |
| 2 | Reduced the number of meals |  |  |  |  |
| 3 | Modified cooking methods |  |  |  |  |
| 4 | Sold assets |  |  |  |  |
| 5 | Borrowed from friends/neigbours/relatives |  |  |  |  |
| 6 | Others (please specify) |  |  |  |  |

58. Food and non-food expenditure in the last four week

| S/N | Foods | Amount | S/N | Expenditure | Amount |
| --- | --- | --- | --- | --- | --- |
| 1 | Rice |  | 1 | Electricity |  |
| 2 | Cowpea |  | 2 | Tv charges |  |
| 3 | Maize |  | 3 | Ceremonies |  |
| 4 | Cassava flour |  | 4 | Religion contribution |  |
| 5 | Garri |  | 5 | Petrol/engine oil/ diesel |  |
| 6 | Yam |  | 6 | Education |  |
| 7 | Yam flour |  | 7 | Kerosene |  |
| 8 | Bread |  | 8 | Gasoline |  |
| 9 | Groundnut oil |  | 9 | Security |  |
| 10 | Palm oil |  | 10 | Membership fees |  |
| 11 | Poultry meat |  | 11 | House rent |  |
| 12 | Beef |  | 12 | Debt repayment |  |
| 13 | Milk |  | 13 | Travel expenses |  |
| 14 | Egg |  | 14 | Clothings |  |
| 15 | Milk |  | 15 | Phone airtime |  |
| 16 | Maize flour |  | 16 | Utility bills |  |
| 17 | Soybean |  | 17 | Personal care |  |
| 18 | Cocoyam |  | 18 | Paper/magazine |  |
| 19 | Fish |  | 19 | Generator |  |
| 20 | Melon |  | 20 | Others, specify |  |
| 21 | Vegetables |  |  |  |  |
| 22 | Tomatoes |  |  |  |  |
| 23 | Onions |  |  |  |  |
| 24 | Pepper |  |  |  |  |
| 25 | Okra |  |  |  |  |
| 26 | Others, specify |  |  |  |  |

59. Indicate the months in the last 10 years. The frequency in which your household encountered food shortage and their severity

| Months in the year in which household encounter serious food shortage | Tick where applicable | Frequency. Rarely= 3, often= 2, usually= 1, others= 0 | Severity. Very severe= 2, severe= 1, less severe= 0 |
| --- | --- | --- | --- |
| January |  |  |  |
| February |  |  |  |
| March |  |  |  |
| April |  |  |  |
| May |  |  |  |
| June |  |  |  |
| July |  |  |  |
| August |  |  |  |
| September |  |  |  |
| October |  |  |  |
| November |  |  |  |
| December |  |  |  |

60. What do you think is the likely cause of food insecurity now?

……………………………………………………………………………………………………………………………………………………………………………………………………………………………………………………………………………………………………………………..
