# Supplementary material for: Improving food security of farming households in Nigeria: Does broiler outgrowers’ program make any difference?
Source: PLoS One. 2023 Sep 21;18(9):e0291611. doi: 10.1371/journal.pone.0291611 (PMC10513292; doi:10.1371/journal.pone.0291611)
Supplement: S1 File — (DOCX) [file pone.0291611.s002.docx]

# Z = $\frac{\mathbf{per} \mathbf{capita} \mathbf{of} \mathbf{nth} \mathbf{household} \mathbf{food} \mathbf{expenditure}}{\mathbf{two}\mathbf{-}\mathbf{third} \mathbf{per} \mathbf{capita} \mathbf{of} \mathbf{all} \mathbf{households} \mathbf{expenditure}}$ Eqn. (1)

$F_{\alpha}= \frac{1}{n}\sum_{i=1}^{q} \left( \frac{z-y_{i}}{z} \right)^{\alpha}$ Eqn. (2)

__ Eqn. (3)

_ Eqn. (4)_

$Y_{i}=\beta_{0}+{\sum_{i}^{9} \beta_{i}X}_{i}+U_{i}$ Eqn. (5)

$\omega_{i}=$E($v_{i}|d_{i})=\frac{-\emptyset(\gamma'Zi )}{1-\theta(\gamma'Zi )}$ Eqn. (6)

E($\omega_{i}|d_{i}=1)$=$\beta_{0}+{\sum_{i=1}^{8} \beta_{i}X}_{i}+\alpha_{i}\omega_{1}$ Eqn. (7)

E($\omega_{i}|d_{i}=0)$=$\beta_{0}+{\sum_{i=1}^{8} \beta_{i}X}_{i}+\alpha_{i}\omega_{0}$ Eqn. (8)
