# Supplementary material for: Improving food security of farming households in Nigeria: Does broiler outgrowers’ program make any difference?
Source: PLoS One. 2023 Sep 21;18(9):e0291611. doi: 10.1371/journal.pone.0291611 (PMC10513292; doi:10.1371/journal.pone.0291611)
Supplement: Attachment — Submitted filename: new_food_security 22-9-22docx no cover page name.docx [file pone.0291611.s003.docx]

Improving food security of farming households in Nigeria, does broiler outgrowers’ programme makes any difference?

**Abstract**

This study profiled the forms of outgrowers’ programme in Osun State with a view to examining the impact of outgrowers’ programme on the food security status of smallholder poultry farming households. Using a primary data survey method, a well-structured questionnaire was designed to collect information from beneficiaries and non-beneficiaries of outgrowers’ programmes in the study area. The data was analysed with the descriptive statistics, food security index and Heckman’s selection model. Descriptive result showed that on the average, the outgrowers and non-outgrowers respectively aged 49 years and 45 years, and both groups respectively had about 12 years and 8 years of experience in poultry farming. About 97% of the outgrowers and 47% of the non-outgrowers belonged to the Poultry Association of Nigeria. ABOP, OBOP, DAYNTEE and GS Farm were the major outgrowers’ programmes practiced in broiler farming. ABOP and OBOP adopted a fixed contract model while DAYNTEE and GSFarm outgrowers’ programmes adopted a semi-fixed contract model. Incidence of food insecurity were 18% for the outgrowers and 35% for the non-outgrowers while the food insecurity depth and severity respectively were 0.025 and 0.033 for the outgrowers and 0.134 and 0.52 for the non-outgrowers. The study showed a significant difference in the perception of food insecurity as well as the coping strategies among the outgrowers and non-outgrowers. Households perceived inadequate resources endowment (MD =0.758, *p*<0.01) and consumption of low cost food (MD=0.0658, *p*<0.01) as the major indicators of food insecurity. The major coping strategies were food acquisition on credit (WMS=1.700), meals adjustment (WMS=1.425), and cooking methods’ modification (WMS=1.875). Probit result revealed that participation in the outgrowers’ programme is influenced by membership of poultry association of Nigeria, credit access and flock sizes. Heckman’s selection model also showed that outgrowers’ participation, household size, gender, marital status and credit access were the significant predictors of food security among the poultry farming households. It was therefore inferred that outgrowers were more food secure than the non-outgrowers, encouraging the need to scale up the programme in the poultry industry. Improvement in the programme awareness and introduction of flexible regulations will make the programmes more rewarding to the beneficiary farming households.

**Keywords:** Anchor Borrowers Scheme, Osun Borrowers Outgrowers’ Programme, Poultry financing policy, food insecurity index, Heckman’s selection model**,** contract farming model; per capita expenditure, SDGs.

**Introduction**

Food is broadly acknowledged as a fundamental need of human life. Food insecurity will exist when all individuals, at all times, are deprived of physical and economic access to adequate, secure and nutritious food that meet their dietary needs and food preference for an active and healthy life (WFS, 1996). According to the Global Hunger Index (2021), about 795 million of the world’s population are chronically undernourished with Africa accounting for more than 60% of the global food insecurity. Nigeria, a major African country in the Sub-Sahara region alone covers approximately 10% of the total food insecure households in Africa having an hunger index score of 28.3 (FAO, 2021; von Grebmer *et al.*, 2021). In comparison to other African counties of similar economies such as Ghana (14.9) and South Africa (12.9), this score portends a serious level of hunger and food insecurity to the nation (von Grebmer *et al.*, 2021). Kuku-Shittu *et al.* (2013) noted that more than 70% of the food insecurity challenge facing Nigeria comes from the rural households where agriculture is the mainstay of the economy. This makes support for agriculture essential for addressing food insecurity in Nigeria.

Being an agrarian society, Nigeria has over 80% of her population employed and earning livelihood in agriculture (Ogunlela and Mukhtar, 2009). The poultry sub-sector, in particular, is occupied by smallholder poultry farming households, rearing birds in the backyards, cages and housings under limited farm technologies and infrastructures (Netherland Agency Enterprise, 2020). These households raise poultry birds with the supports of other members living and pooling resources together and as well eating from the same pot under the same roof (Babatunde *et al.,* 2007). Poultry farming households plays significant roles in realizing the sustainable development goal of zero hunger in Nigeria by producing meat and eggs for essential proteins and earning income to sustain farming households, however, the system is bedeviled with a myriads of challenges including poor financing and market price volatility which limits the capacity of the farming households to be food secure (Liverpool-Tasie *et al*., 2017; Vaarst *et al.,* 2015; Bature *et al.,* 2013; Abu and Soom, 2016; Olounlade *et al.,* 2021).

Outgrowers’ programme is one of the market-based financial initiatives to arrest the rising business uncertainties, improve farm income, socioeconomic status and food security of the broiler farmers in Nigeria (CBN, 2016; Odunlanmi *et al.,* 2016; Adereti and Fadare, 2017). In other words, outgrowers’ programme is a form of contract farming aimed at addressing the lingering problems of broiler farm financing and marketing in Nigeria (Minot, 2011). The programme is designed to provide birds, cash, feeds, and market supports to broiler farmers, and subsequently establishes a linkage between smallholder poultry farmers (out-growers) and reputable large scale processors (off-takers) for the overall enhanced capacity utilization of the coordinated industries. Outgrowers’ contract agreement obliged the off-taker firm to cover the working capital and the running cost of raising broiler birds for a pre-determined period, usually 6 weeks, and off-take the resulting birds at the pre-agreed weight, price, and quantity. As a prerequisite for participation, this programme targets existing broiler farmers that have affiliations with the Poultry Association of Nigeria and the capacity to meet the required structures for broiler production.

The design of outgrowers’ programme allows individuals, group of individuals and State governments to anchor the programme (Bijman, 2008). Osun State happens to be one of the States in Nigeria where both the private, State and Federal forms of outgrowers’ programmes run concurrently in the Nigeria’s poultry sub-sector. These programmes, refers to as Osun broilers’ outgrowers programme (OBOP) and anchor-borrowers’ programme (ABP), have been in practice in the State since 2011 and 2018 respectively. There is an evidence that in the last three years of OBOP operation, approximately 162 poultry farmers have benefited with a total capital investment of about ~~N~~788 million, producing over 5 million birds (Emovwodo, 2019).

Several studies have investigated the impact of outgrowers programme in Nigeria with mixed outcomes. Bature *et al.* (2013) and Olounlade *et al.* (2020) noted that the outgrowers have a higher tendency to be food insecure than the non-outgrowers as the later mostly take advantage of fluctuations in the market prices. There are further arguments that the terms of the contract play a significant role in determining the welfare impact of the programme (Ani, 2017). On the other hand, studies conducted by Ani (2017); Sanusi (2017); Abdulazeez *et al.* (2018) and Adetomiwa *et al.,* (2020) asserted that farmers participating in outgrowers’ programme had higher income and were more likely to be food secure than the non-participating farmers. With several works of literature on the impact of outgrowers programme focusing on the crop sector, there is a dearth of knowledge on the impact of outgrowers programme on food security of the poultry farming households in Nigeria. To this effect, we seek to explore the impact of the outgrowers’ programme on food security of smallholder poultry farming households with a view to describing socio-economic characteristics of broiler outgrowers and non-outgrowers; profiling forms and contract models for outgrowers’ programmes existing in the State; evaluating food security status of the broiler outgrowers and non-outgrowers, describing food insecurity perception and coping strategies among poultry outgrowers and non-outgrowers’ households, and determining effect of participation in poultry outgrowers’ programme on food security of participating smallholder poultry farmers in the study area.

**Materials and Methods**

This study was carried out in Osun State, Southwestern Nigeria. The State is located between latitudes 5º58’N and 8º07’N of the equator and longitude 4º00 E and 05º05’E of the Green meridian and has a land area of about 9,251km with a population of 3,416,959 people (NCP, 2006). Osun State has 30 Local Governments Area with three agricultural zones which are Iwo, Osogbo and Ife/Ijesha. It is bounded in the north by Kwara State, in the east partly by Ondo and Ekiti States, in the south by Ogun State and in the west by Oyo State. The State has a tropical humid climate with its characteristics temperature all year round, the dry season occurs between November and March while the rainy season occurs between April and October with a mean annual rainfall of about 1,000 mm and a mean annual temperature of 21.1°C to 31.1°C that favours poultry farming. At the first quarter of 2022, there are about 42 and 105 beneficiaries of ABP and OBOP in Osun State according to the report from Osun State Ministry of Agriculture and Central Bank of Nigeria. Some of these farmer participated in 2 or more outgrowers’ programmes at a time which were mostly OBOP and ABP.

We deployed a multistage sampling technique to collect primary data for this study using a well-structured questionnaire. At the first stage, we purposively selected broiler farmers because the poultry-based outgrowers’ programme targeted only the broiler farmers in Nigeria. At the second stage, we obtained the list of beneficiaries of the outgrowers programme from the Central Bank of Nigeria and the Ministry of Agriculture, Osun State. At the third stage, using a random sampling procedure, we selected six Local Government Areas (LGAs) from the State based on the prevalence of broiler farmers in the area. Then at the fourth stage, from each of the six Local Government Areas, we selected 10 outgrowers and 10 non-outgrowers randomly making a total of 60 outgrowers and 60 non-outgrowers. However, only 40 outgrowers and 60 outgrowers interviewed had sufficient information for statistical analysis. Data collection for the study covered a period of 4 months between April 2021 and July, 2021. Data obtained was based on broiler farm production activities during the first quarter of 2021. From the data gathered, we obtained information on the socioeconomic characteristics of smallholder poultry farmers such as gender, age, household size and level of education; types of outgrowers’ programmes; bird capacity and flock size, broiler production cost and income; and perception and coping strategies for food insecurity; food expenditure. We analysed the data obtained using descriptive statistics, Foster-Greer-Thorbecke (FGT) food security index and the Heckman’s selection model.

We used descriptive statistics including frequency count, percentage, and arithmetic mean and Figure 1: the map of Osun State showing the LGA. ([your jigsaw puzzle](http://www.yourjigswpazzle), 2022)

weighted mean score to identify the socioeconomic characteristics of the respondents and profile forms of outgrowers’ programme, as well as the food security perception and coping strategies among broiler farmers.

**Weighted Mean Score (WMS)**

To profile food security perception and coping strategies of broiler farming households, we attached **w**eighted points to a 3-point Likert scale as presented: very often (VO)= 3 points; Regular (R) = 2 points; Occasional (O) =1 point; Never (O)=0 point; Then we calcated the weighted mean score for each item ith as

WMS=$\frac{[3\left( n_{3} \right)+2\left( n_{2} \right)+1\left( n_{1} \right)}{N}$	(6)

Where $n_{1}-n_{3}$ = are frequencies of Likert options for ith item, N=total number of respondents.

We also determined the average mean as the ratio of number of options to the total options (3)

Average = $\frac{[3+2+1]}{3}$ = 2.0

Thus, we regarded any item with a 2.0<WMS$\leq$3.0 as a chronic food insecurity situation; items with a 1.0<WMS$\leq$2.0 as an occasional food insecurity while items with a WMS$\leq$1.0 as a break-even food security situation.

**Food security index model Specification**

Using the food security index model, we determined the food security index of outgrowers and non-outgrowers by estimating the per capita household food expenditure. Following Adetomiwa *et al.* (2020) and Adepoju and Adejare,( 2013), we derived the food security index by dividing the per capita n^th^ household food expenditure by two-thirds mean per capita of all the households’ expenditure as given in Eqn (1).

Z = $\frac{per capita of nth household food expenditure}{two-third per capita of all households expenditure}$	Eqn. (1)

Where Z = Food security index/line

If Z ≥ 1, the household is food secure

If Z ≤ 1, the household is food insecure

Therefore, we categorized households whose per capita monthly food expenditure were at least two-third of the mean per capita monthly food expenditure as food secure. However, we regarded those households whose per capita monthly food expenditure were less than two-third of the mean monthly per capita food expenditure as the food insecure households. We further employed the Foster, Greer and Thorbecke index used by Adetomiwa *et al.* (2020) and Adepoju and Adejare (2013) to determine the food security index and the depth, gap and severity. The model is given as

$F_{\alpha}= \frac{1}{n}\sum_{i=1}^{q} \left( \frac{z-y_{i}}{z} \right)^{\alpha}$	Eqn. (2)

Where:

F= food security measure (incidence, depth, severity)

$\alpha=poverty aversion parameters, it takes values of 0, 1 or 2$

n = total number of households in the population

q = number of households below the food security line (head count)

$y_{i}=per capita food expenditure in increasing order for all households$

z = food security line$(\mathrm{mean}\frac{2}{3} per capita of all households expenditure)$

**Heckman’s selection model**

We employed Heckman’s selection model to analyse the impact of outgrowers’ programme participation on household food security. We considered this model suitable due mainly to its capacity to correct for the non-random bias associated with the selection of poultry farmers for the outgrowers’ programmes. According to Hecknan (1988), the Heckman’s selection model is a two-stage procedure. We employed probit model at the first stage to determine the factors influencing participation of poultry farming households in the outgrowers programme. The model is given as

Eqn. (3)

Where d*_i_* is the participation status of household; di = 1, for the outgrowers and 0 for the non-outgrowers. Ф(.) is the cumulative standard normal distribution; Z_i_ is the observable socio-economic variables of the farming households including PAN membership, flock size, household head experience, age and gender of the household head, and $\gamma'$ is the the coefficient of predictor variable Z_i ._

The mode has a likelihood function

 _Eqn. (4)_

Where di is then estimated and inserted into the main welfare equation capturing food securing. As required by Heckman’s selection model, at the second stage, we employed ordinary least square model to determine the effect of the outgrowers’ programme participation on the food security of the outgrowers. The model is given by

$Y_{i}=\beta_{0}$+${\sum_{i}^{9} \beta_{i}X}_{i}+U_{i}$	Eqn. (5)

Where:

$Y_{i}$ = food security index (two-third annual per capita household expenditure); $X_{1}=household size$ (count);$X_{2}=$marital status (1=married, 0=otherwise);$X_{3}=PAN membeship; X_{4}=household head experience in poultry (years); X_{5}=flock size (count); X_{6}$= bird selling price (naira); X_7_ = household head access to credit facility (1=yes, 0=otherwise); X_8_ =Bird type (1=broilers only, 0=combined); $X_{9}$=$gender of household head(1= male, 0= female)$; $U_{i}$ = random error term; $\beta_{0}$= constant term.

In order to account for the selectivity bias, we estimated Inverse Mills Ratio (Heckman, 1988) which correct for the correlation between the error term and the dummy variable by incorporating the expected value of selection error into the equation of the welfare outcome (food security). This is obtained by

$\omega_{i}=$E($v_{i}$|$d_{i})=\frac{-\emptyset(\gamma'Zi )}{1-\theta(\gamma'Zi )}$	Eqn. (6)

We then computed and fitted the Inverse Mill Ratio ($\omega_{i}$) in the OLS regression model from which the welfare equation becomes

E($\omega_{i}$|$d_{i}=1)$=$\beta_{0}$+${\sum_{i=1}^{8} \beta_{i}X}_{i}+\alpha_{i}\omega_{1}$	Eqn. (7)

E($\omega_{i}$|$d_{i}=0)$=$\beta_{0}$+${\sum_{i=1}^{8} \beta_{i}X}_{i}+\alpha_{i}\omega_{0}$	Eqn. (8)

Where $d_{i}$=1 if the household participated in outgrowers’ programme and $d_{i}$=0 if otherwise.

**Results and Discussion**

**Socio-economic characteristics of the outgrowers and non-outgrowers of outgrowers’ programme**

As presented in Table 1, the age distribution of poultry household heads in the study area shows that the outgrowers and non-outgrowers had a mean age of 49.38±9.15 years and 45.60±9.39 years respectively. Clearly, household head in both groups shared a similar average age, suggesting that both the outgrowers and non-outgrowers were in their active and economic age and that outgrower programme does not seems to favour certain age category. This finding is in line with Abu and Soom (2016) who noted that poultry farmers were primed-aged individuals probably as a result of the tedious and tasking nature of poultry activities. In terms of gender distribution, data in Table 1 reveals male dominance in poultry farming as 78% and 72% of the households for the outgrowers (WMS=0.72$\pm$0.45) and the non-outgrowers (WMS=0.78$\pm$0.42) were headed by male gender. The implication is that poultry outgrowers’ programmes are not gender biased in the study area. The submission disagrees with the study of Odunlami *et al.* (2016) that developmental programmes and loans interventions favour male farmers in Lagos State. Thus, outgrowers’ programme provides a level playing ground for both gender in Osun State. Marital status averaged 1.97$\pm$0.42 for the outgrowers and 2.03$\pm$0.55 for the non-outgrowers indicating dominance of married households in broiler farming. This result agreed with findings of Odunlami *et al.* (2016) that majority of individuals engaging in farming operations are married. The outgrowers and the non-outgrowers had a mean household size of 6.48±1.74 members and 5.52±2.40 members respectively. Interestingly, we can say that households in the study area have a considerable number of hands to feed and carter for, and n thereby, have high tendencies to be exposed to the problem of food insecurity. Data in Table 1 also reveals that both the outgrowers and non-outgrowers respectively spent on the average, 11.75±6.32 and 8.23±5.36 years in schooling. This suggests that majority of the outgrowers have a minimum of secondary school degree certificate, whereas, the non-outgrowers were mainly primary school degree holders. Thus, education may likely play an important role in outgrowers’ programme participation in Osun State. This submission agrees with Adereti and Fadare (2017) and Babatunde (2007) that education opens the minds of farmers to participate in various agricultural development programmes in Nigeria. The mean years of experience for the outgrowers was 11.7±6.32 years whereas, the non-outgrowers had 8.23±5.34 years, however, both groups were not significantly different in poultry experience (t=0.21, *p*>0.1). Notwithstanding, the majority of the outgrowers had been involved in the raising of broiler birds for over 10 years. This result validates importance of experience as a necessary condition for participation in the outgrowers’ programme. Membership of trade association in Table 1 data shows that 98% of the outgrowers and 47% of the non-outgrowers were members of the poultry association of Nigeria (PAN). The finding indicates that being a member of PAN is a basic requirements for participating in the outgrowers’ programmes. This is in line with Adetomiwa *et al.,* (2020). Table 1 further revealed that 83% of the outgrowers and 52% of the non-outgrowers had access to credit, with the majority of both groups respectively acquiring credit mainly from co-operatives groups. This agrees with the finding of Babatunde *et al.* (2007) that members of co-operative groups had more credit access, and promotion of co-operatives groups should be of utmost concern by government and promoters of economic interventions.

**Table 1**: **Socioeconomic Characteristics of the smallholder poultry households**

| Variables | outgrowers | | Non-outgrowers | |
| --- | --- | --- | --- | --- |
|  | Mean | Std. Dev | Mean | Std. Dev |
| Age  Gender  Marital status  Household size.  Level of education  Year of experience  Poultry Association of Nigeria  Access to credit  Credit source | 49.38  0.78  1.97  6.48  6.87  11.75  0.98  0.83  5.12 | 9.15  0.42  0.42  1.74  1.41  6.32  0.16  0.38  3.09 | 45.60  0.72  2.03  5.52  5.90  8.23  0.47  0.52  4.48 | 9.39  0.45  0.55  2.40  2.22  5.34  0.50  0.51  1.48 |

Source: Author’s field survey, 2021.

**Forms and bird releasing capacity of Outgrowers programmes in Osun State**

From the data presented in Figure 2, we identified four major forms of outgrowers’ programmes in Osun State: Anchor Borrowers’ Outgrowers’ Programme (ABOP), Osun State Broiler Outgrowers’ Programme (OBOP), DAYNTEE farm outgrowers’ programme, and GS agricultural limited outgrowers’ programme. About 30% of the outgrowers took part in the ABOP, 48% participated in the OBOP, 15% took part in the GS agricultural limited outgrowers’ programme, while 8% participated in the DAYNTEE farm outgrowers’ programme. The large involvement in government-sponsored outgrowers’ programmes (ABOP and OBOP) is attributable to the mass awareness and intensive public sensitization via social networking sites and the involvement of Poultry Association of Nigeria (Emovwodo, 2019).

proportion of outgrowers involved (%)

| Forms of outgrowers’ programmes | Frequency |
| --- | --- |
| ABOP | 30% |
| OBOP | 48% |
| DAYNTEE | 8% |
| GSFARM | 15% |

Figure 2: Distribution of respondents by the existing forms of poultry outgrowers’ programmes and involvement rate in Osun State

Data in Table 2 also shows the bird releasing capacity, contract models and sponsors of outgrowers’ programmes available in Osun State. We found out that ABOP and OBOP released fixed quantity of broiler birds. In terms of bird releasing capacity, ABOP issued 1,000 day old chicks while OBOP released 2,000 day old chicks. Conversely, GS Farms and DAYNTEE released varied quantity of broiler birds. GS Farms issued between 1,000 - 5,000 day old chicks while DAYNTEE released a minimum of 2,000 day old chicks which could rise to over 5,000 day old chicks based on the carrying capacity of the outgrowers. In terms of sponsorship, we discovered that ABOP and OBOP were driven and financed by Federal and Osun State Governments respectively. Both programmes had a rigid financing policy modeled as a capsule. The outgrowers under these contracts do not have any prerogative right to choose the input items to receive from the sponsors. DAYNTEE and GS Farms on the other hand operated a flexible outgrowers’ contract model. Both programmes were semi-flexible in bird supply basically because it allowed outgrowers to make decision on the bird quantity to receive but could not has no capacity to influence the quantity of complementary inputs provided under the scheme. Moreover, the anchor group has the exclusive right to decide the equivalent quantity of the complementary inputs that will be released to the outgrowers. Thus, GS farm and DAYNTEE outgrowers’ programmes operated a non-capsule policy model. Exclusively, GS Farms outgrowers’ programme further expands their coverage and strengthens the firm’s position in the poultry industry by off-taking broiler birds that have 2.0kg weight and above from the non-beneficiaries of their programme. The implication is that DAYNTEE and GS Farm contract models are more likely to be attractive to poultry farmers than the ABOP and OBOP models, resulting to the possibilities of losing beneficiaries of OBOP and ABOP to the private driven outgrowers’ programmes in the nearest future. This does not portent a good future for the government driven outgrowers’ programmes in Nigeria.

Table 2: Types of outgrowers in broiler production in Osun State

| Broilers’ outgrowers type | Bird Capacity (count) | Contract Model | Sponsors |
| --- | --- | --- | --- |
| ABOP | 1,000 | fixed | Federal Government |
| OBOP | 2,000 | fixed | State Government |
| GS Agriculture limited | 1,000-5000 | semi-flexible | private |
| DAYNTEE Farm | 2,000-5,000+ | semi-flexible | private |

**Food Security of the outgrowers and Non-outgrowers’ households**

Data in Table 3 reveals the food security indices of the outgrowers and non-outgrowers in the study area. The food security line was ₦11,098.59 for the outgrowers and ₦10,188.97 for the non-outgrowers. From the data in Table 3, we found out that the majority of the outgrowers (82.5%) and non-outgrowers (65.0%) were food secure. The food security status of the outgrowers was significantly different (t-statistics= 2.013, *p*<0.05) from the non-outgrowers, indicating that the households of the outgrowers are more food secure than the households of the non-outgrowers in Osun State broiler farming. This submission supports findings of Ani (2017), Sanusi (2017) and Abdulazeez *et al.* (2018) who found out that outgrowers have a higher tendency to be food secure but contradicts reports by Bature *et al.* (2013) and Olounlade *et al.* (2021) that outgrowers’ programme exploit and does not improve food security status of the beneficiaries because non-outgrowers take advantage of fluctuations in market prices to better their position in the market.

Table 3: Distribution of food security status of the outgrowers and non-outgrowers in the poultry outgrowers’ programme

| Categories | Food Secure | | Food Insecure | | Per capita household food expenditure (₦) | Total |
| --- | --- | --- | --- | --- | --- | --- |
|  | Frequency | % | Frequency | % |  |  |
| Outgrowers | 33 | 82.5 | 7 | 17.5 | 11,098.59 | 40(100) |
| Non-outgrowers  t-statistics | 39  2.013 | 65.0 | 21  1.06 | 35.0 | 10,188.97 | 60(100) |

Furthermore, we realized from data in Table 4 that the incidence of food insecurity was 0.175 for the outgrowers and 0.350 for the non-outgrowers. This indicates that 17.5% of the outgrowers and 35.0% of the non-outgrowers had their per capita expenditure on food below the food security line. The depth of food insecurity was 0.025 for the outgrowers and 0.134 for the non-outgrowers. This implies that the food insecure households among the outgrowers and non-outgrowers respectively will need to expend an additional 2.5% and 13.4% of the household income on food to escape food insecurity. In addition, the severity of food insecurity was 0.033 for the outgrowers and 0.052 for the non-outgrowers. The results obtained from Table 5 implies that food insecurity indices were lower among the outgrowers than the non-outgrowers. Interestingly, this may be a proof of the impact of the outgrowers’ initiative in reducing the food insecurity among the farmers. However, this result suggests that the outgrowers programme is yet to totally lift all the poultry participating household from food insecurity in Osun State.

Table 4: Food insecurity indices for the outgrowers and non-outgrowers

| Categories | Food Insecurity Indices | | |
| --- | --- | --- | --- |
|  | Incidence (F_0_) | Depth (F_1_) | Severity (F_2_) |
| outgrowers | 0.175 | 0.025 | 0.033 |
| Non-outgrowers | 0.35 | 0.134 | 0.052 |
| t-test | 1.06 | 0.39 | 1/09` |

**Food insecurity perceptions among poultry households**

Data in Table 5 reveals the perception of the households about their food insecurity status. Generally speaking, in the past 4 weeks, both the outgrowers (WMS=1.250±0.494) and the non-outgrowers (WMS=1.412±0.530) opined that they occasionally did not have enough food to eat (mean difference=0.167, t=1.040). Similarly, we found out that both the outgrowers (WMS=1.025±0.158) and the non-outgrowers (WMS=1.133±0.343) occasionally had their households skipped a meal because there was not enough money to buy food (mean difference=0.108, t=0.397). Table 5 also reveals that both the outgrowers (WMS=1.125±0.335) and the non-outgrowers (WMS=1.183±0.390) occasionally ate smaller meal than their households felt they needed (mean difference=0.058, t=0.143). However, respondents affirmed that in the past four weeks, both the outgrowers (WMS=1.00±0.000) and the non-outgrowers (WMS=1.017±0.129) did not go a whole day and night without eating (mean difference=0.167, t=1.000).

On the other hand, the outgrowers differed significantly in mean difference from the non-outgrowers on their perception of eating of kinds of food, unwanted foods, and low cost foods in the last 4 weeks. The report in Table 5 shows that outgrowers (WMS=1.375±0.638) opined that their households occasionally unable to eat kinds of food due to lack of resources whereas, this assertion was perceived to be very regular among the non-outgrowers (WMS=2.133±0.812), suggesting the possibility of a chronic food insecurity situation due mainly to inadequate household resource endowment among the non-outgrowers. This submission was supported by a mean difference and t-value of 0.758 and -4.035 for both groups. In like manner, the outgrowers (WMS=1.530±0.599) reported occasional consumption of foods items that they really did not want to eat due mainly to lack of resources to obtain other type of food, whereas, the non-outgrowers (WMS=1.933±0.686) asserted that lack of resources to obtain other types of food sometimes compelled their households to eat food items they did not really want to eat. The mean difference (0.408) of the outgrowers over the non-outgrowers was significant (t=1.732) indicating that inadequate household resources forced non-outgrowers to eat food items they did not really desire. In the last 4 weeks, the outgrowers (WMS=1.375±0.638) also noted that their household occasionally rely on any kind of low cost food to feed. The non-outgrowers on the other hand, believed that they often rely on any kind of low cost food to feed (WMS=1.833±0844). The mean difference (0.658) of the outgrower over the non-outgrowers significant (t= -3.761), pointing to an evidence of chronic food insecurity among the non-outgrowers.

**Coping mechanisms against food insecurity among poultry farming households**

Table 6 indicated that the major coping strategies against food insecurity among the participating households was the modification of cooking methods (WMS=1.875±0.883) and purchase food on credit (WMS=1.700±0.853). This practice was common to 62% of the outgrowers. In all, 45% of the outgrowers modified cooking methods occasionally (45.0%), 10% on regular basis while 7% did so very often. Also, about an half of the outgrowers representing 47% each obtained food on credit (WMS=1.700±0.853) and borrowed from friends and relatives (WMS=17.00±0.911). Among these outgrowers, 27% obtained food on credit occasionally and 17% others got food on credit on regular basis. Similarly, 32% occasionally borrowed cash from friends and relatives; 7% each borrowed from family and relatives on very often and regular basis. A few outgrowers (WMS=1.425±0.712) representing 32% reduced their daily meals. Of the percentage, about 25% alone did so on occasional basis. However, the practice of sales of asset was uncommon among the participating households (WMS=1.325±0.724) representing 20% of the outgrowers.

The reverse is the case among the non-outgrowers. Result in Table 6 reveals that about 68% obtained food on credit (WMS=1.360±0.637) out of which 7% were very often, 17% were regular and 45% were obtained occasionally. About 82% reduced their number of meals per day (WMS=1.360±0.489). Among those that reduced daily meal intake, 13% experienced this very often, 35% on regular basis while 33% were occasional. Similarly, 80% of the non-outgrowers modified their cooking methods (WMS=1.480±0.653). In all, 25% modified cooking habit very often, 28% did so on regular basis while 27% modified cooking on occasional basis. Borrowing from friends and relatives (WMS=1.320±0.557) was also one of the major approaches non-outgrowers adopted to cope with food insecurity. However, the sales of household asset (1.000±0.000) was not a common practice among the non-outgrowers as 25% out of the 33% households that sold asset did so on occasional basis. Out of the 67% households that borrowed from friends and relatives, 43% practiced this occasionally while 15% borrowed on regular basis.

**Table 5: Households’ perception of food insecurity**

| Four weeks food security perception | Outgrowers | | Non-outgrowers | | Mean difference | t-statistics |
| --- | --- | --- | --- | --- | --- | --- |
|  | Weighted mean | Remark | Weighted mean (S.D.) | Remark |  |  |
| Have enough to eat | 1.250 (0.494) | occasional food insecure | 1.412 (0.530) | occasional food insecure | 0.167 | 1.04 |
| not able to eat kinds of food due to lack of resources | 1.375 (0.638) | occasional food insecure | 2.133 (0.812) | chronic food insecurity | 0.758 | -4.035* |
| eat unwanted foods items due to lack of resources to obtain other food types | 1.53 (0.599) | occasional food insecurity | 1.933 (0.686) | occasional food insecurity | 0.408 | -1.732** |
| skip a meal due to insufficient money to buy food | 1.025(0.158) | occasional food insecurity | 1.133 (0.343) | occasional food insecurity | 0.108 | -0.397 |
| eat smaller meal than felt needed | 1.125 (0.335) | occasional food insecurity | 1.183 (0.390) | occasional food insecurity | 0.058 | 0.143 |
| rely on any kind of low cost food to feed | 1.375 (0.638) | occasional food insecurity | 2.033 (0.844) | Chronic food insecurity | 0.658 | -3.761* |
| Went a whole day and night without eating | 1.000 (0.000) | break even food security | 1.017 (0.129) | occasional food insecurity | 0.0167 | -1.00 |

Source: Field Survey, 2021	*, **, ***=significant at *p*<0.01, *p*<0.05, *p*<0.1 respectively

**Table 6: Coping mechanisms used against food security by poultry farmers**

| Coping Mechanisms | Outgrowers (n= 40) | | | | | Non-outgrowers (n= 60) | | | | |
| --- | --- | --- | --- | --- | --- | --- | --- | --- | --- | --- |
|  | Very often (%) | Regular (%) | Occasional (%) | Never  (%) | WMS | Very often (%) | Regular  (%) | Occasional (%) | Never  (%) | WMS |
| Obtained food on credit | 2.50 | 17.50 | 27.50 | 52.50 | 1.700  (0.853) | 6.67 | 16.67 | 45.00 | 31.67 | 1.360  (0.637) |
| Reduced number of meals | 2.50 | 5.00 | 25.00 | 67.50 | 1.425  (0.712) | 13.33 | 35.00 | 33.33 | 18.33 | 1.360  (0.489) |
| Modified cooking methods | 7.50 | 10.00 | 45.00 | 37.50 | 1.875  (0.883) | 25.00 | 28.33 | 26.67 | 20.00 | 1.480  (0.653) |
| Sold asset | 5.00 | 2.50 | 12.50 | 80.00 | 1.325  (0.764) | 1.67 | 6.66 | 25.00 | 66.66 | 1  (0.00) |
| borrow from friends and relatives | 7.50 | 7.50 | 32.50 | 52.50 | 1.700  (0.911) | 5.00 | 15.00 | 43.33 | 33.33 | 1.32  (0.557) |

Source: Field Survey, 2021

**Determinants of households’ participation in outgrowers’**

Table 7 presents the determinants of household participation in outgrowers’ programmes. As shown in the result, PAN membership, credit access and flock sizes significantly (β=1.923, *p*<0.000; β=0.604 *p*<0.1; β=0.001 *p*<0.01) increased the likelihood of participating in the outgrowers’ programmes.

The result shows that if there is a 1% increase in PAN membership, the likelihood that a broiler farmer will participate in the outgrowers’ programmes will increase by 192.3%, meaning that PAN membership is an important factor in outgrowers’ participation. It is therefore necessary that poultry farmers are encouraged to join PAN to increase the chance of participating in the programme. This finding supports Adetomiwa *et al.* (2020) who reported the importance of PAN membership in outgrowers programme’s participation. Similarly, flock size was positively correlated to outgrowers’ participation. If there is a 1% increase in the flock size of broilers’ farms, participation in outgrowers’ programmes will likely increase by 0.1%. In addition, positive relationship exists between access to credit and participation in outgrowers’ programmes. With a 1% increase in credit access, the livelihood that a broiler farmer will participate in the outgrowers’ programmes will rise by 60.4%. This means that outgrowers’ programmes is more favourable to the broiler farmers that have access to credit than those without it. Abu and Soom (2016) noted the importance of credit access to farm. This study thus affirmed that outgrowers’ programmes provide support for farmers who have production facilities but lack the running cost of raising birds to maturity. This is an indication that broilers’ outgrowers programme is fulfilling its aim of financing and commercializing smallholder poultry farming in Nigeria.

**Effect of outgrowers programmes on per capita food expenditure of outgrowers.**

The estimate of the Heckman’s selection model in Table 8 shows the relationship between the households’ socioeconomic variables, outgrowers variables and per capita food expenditure (food security) of the outgrowers of outgrowers’ programmes. The result shows that outgrowers’ participation; gender of household head, marital status and credit access significantly (β=0.091, *p*<0.01; β=0.248, *p*<0.05; β=0.152, *p*<0.1 and β=0.214, *p*<0.1) increased food security while household size significantly (β= -0.245, *p*<0.01) reduced it.

Table 8 also shows a positive correlation between gender of household head and per capita food expenditure. If household head’s gender changed by 1% (from female to male for instance), household per capita food expenditure will improve by 24.8% indicating that men spent more than women on food items and as such, male headed households are more food secure than the female headed households in outgrowers’ programme. A study by Adetomiwa *et al.* (2020) also reported similar result. This finding indicates that female headed households involved in outgrowers’ programme are predisposed to food insecurity despite the intervention. Thus, it may be necessary to explore the gender composition of beneficiaries of outgrower’s programmes in Osun State.

Similarly, access to credit exerts positive influence on household food security. If there is 1% increase in access to credit, there will be a corresponding 7.5% improvement in households’ per capita food expenditure, indicating that outgrowers that have access to credit probably employ credit in productive ventures as such, are more food secure than those without it. The reasons for this is not far-fetched as outgrowers’ programme provides a favourable platform to expand farm investment to the outgrowers. This corroborate with Adereti and Fadare (2017) on the benefits of developmental programmes to the farming households. The result in Table 7 also reveals that 1% increase in marital status will result to about 10.2% boost in the per capita food expenditure of the participating households. This indicates that outgrowers that are married and have additional responsibilities are more food secure than the single outgrowers.

On the other hand, household size had a negative correlation with food security. The Heckman’s model shows that a 1% increase in household size will decrease the per capita food expenditure of the outgrowers by 24.5%. This implies that large household outgrowers will struggle to be food security under the programme. The report corroborate findings by Babatunde (2007) that household with large sizes are more likely to be food insecure. We realized that despite the positive correlation between marital status (married) and food security, there is a threshold household size that guarantees the food security of outgrowers. There is no gainsaying that large households exert pressure on the limited household resources, as a result, large household outgrowers need to engage their household members in productive ventures to sustain household food security. Interestingly, it may be necessary that the outgrower trim their household size to reap the food security reward of outgrowers’ programme in Nigeria’s poultry industry. This submission supports Babatunde (2007) whose finding shows that household size is a key determinant of food security among the rural households. On the other hand, marital status had a positive correlation with food security.

The negative correlation between participation (IMR) and food security indicates that there is a significant negative selectivity bias among households. A negative selectivity bias as interpreted by Adetomiwa *et al.* (2020) shows that outgrowers experienced higher level of food security than outgrowers. Thus, mean per capital expenditure of outgrowers will increase by 91% due to participation in outgrowers’ programme. This further prove that outgrower programme is an effective tool to raising food security among poultry farmers in Nigeria.

Table 7: Heckman’s selection model showing determinants of outgrowers’ participation and its effect on household food security

| Variables | Selection/Determinants | | Outcome/Impact | |
| --- | --- | --- | --- | --- |
|  | Marginal effect | p-value | Coef. | p-value |
| Household size | -0.072 | 0.466 | -0.245 | 0.000*** |
| Marital status | -0.437 | 0.156 | 0.152 | 0.102* |
| PAN membership | 1.923 | 0.00*** | 0.016 | 0.958 |
| Years of experience | -0.029 | 0.421 | -0.005 | 0.639 |
| Flock size | 0.001 | .001*** | 0.0008 | 0.743 |
| Flock selling price | -0.001 | 0.449 | 0.0007 | 0.441 |
| Credit access | 0.604 | .081* | 0.214 | 0.075* |
| Age of the household head | 0.476 | 0.659 |  |  |
| Level of education | 0.069 | 0.489 |  |  |
| Bird type |  |  | -0.038 | 0.647 |
| Gender |  |  | 0.248 | 0.039** |
| IMR (participation) |  |  | 0.91 | 0.027*** |
| Constant | -2.286 | 0.468 | 3.282 | 0.003*** |
| athrho | -.78 (0.912) | 0.392 |  |  |
| lnsigma | -1.2 (0.311) | 0.000*** |  |  |
| Mean dependent var 0.400 SD dependent var 0.92 Number of observations 100 Chi-square Prob > chi2 0.000 . Akaike Crit. (AIC) 134.691 | | | | |
| *** indicates a significant level at 1%, **indicates a significant level at 5%, * indicates a significant level at 10% | | | | |

**Conclusion and recommendations**

The study evaluated the impact of outgrowers’ programmes on the food security of outgrower farming households in Osun State, Nigeria. Participation in outgrowers’ programmes is mainly influenced by the compulsory membership of poultry association of Nigeria, farmers access to credit and availability of farm infrastructure with bird capacity that meets the stated number of birds by the programmes. We found out that outgrowers were more food secure than the non-outgrowers and food security of outgrowers is strongly influenced by participation in outgrower programme, gender and marital status of the household head, household size, and credit access. However, there is a threshold household size that guarantees food security of outgrowers. We further discovered among the outgrowers that the female headed households are predisposed to food insecurity despite the intervention in the poultry industry.

I. In the light of the fact that outgrowers programme increases food security among broiler farmers, developmental programmes in the nearest future should be designed in such a way to accommodate more smallholder farmers. Farmers who lack the structure to produce birds but have experience in poultry production should be put into consideration
II. Since household size was a critical factor in the food security model, family planning encouraged and reproductive education should be introduced among the farming households
III. Smallholder farmers should be encouraged to join the poultry association as it was found to be a criterion to participate in outgrowers programmes and it was observed that farmers belonging to poultry associations had more access to information and extension agents.

**References**

Abdulazeez, R.O., Musa, M.W., Saddiq, N.M., Abdulrahman, S. and Oladimeji, Y.U. (2018). Food Security Situations among Farmers under Kogi Accelerated Rice Production; a USDA Approach. Journal of Agricultural Economics, Extension and Social Sciences, 1(1)23-130

Abu, G.A. and Soom, A. (2016). Analysis of factors affecting food security in rural and urban farming households of Benue state, Nigeria. *International Journal on Food Agricultural Economics*, 4:55–68.

Adepoju, A.O. and Adejare, K.A. (2013). Food Insecurity of Rural Households during Post-Planting Season in Nigeria. *Journal of Agriculture and Sustainability*, 4(1):16-35.

Adereti, F.O. and Fadare, I.A. (2017). Role of Fadama III Project in Improving the Socio-Economic Status of Rural Dwellers in Osun State, Nigeria. *International Journal of Agriculture, Forestry and Plantation,* 5(6):66-71

Adetomiwa, K., Mayowa, O.O., Adebayo, A.I. and Victor, O.O. (2020). Impact Assessment of Fadama III Group Participation on Food Security Status of Rural Households in South West, Nigeria. *Journal of Agriculture and Sustainability*, 13:1-29.

Ani, N.M. (2017). Impact of the Commercial Agriculture Credit programme on the Performance of Beneficiaries in Anambra State Nigeria.

Babatunde, R.O., Omotesho, O.A. and Sholotan, O.S. (2007). Socio-Economics Characteristics and Food Security Status of Farming Households in Kwara State, North-central Nigeria. *Pakistan Journal of Nutrition,* 6:49-58. [ONLINE] Available at http://dx.doi.org/10.3923/pjn.2007.49.58

Bature, M.Y., Sanni A.A., and Adebayo F.O. (2013). Analysis of Impact of National Fadama Development Projects on Beneficiaries Income and Wealth in FCT, Nigeria. *Journal of Economics and Sustainable Development,* 4(17):86-91.

Bijman, J. (2008). Contract farming in developing countries: An overview. Working Paper, Wageningen University.

Central Bank of Nigeria, Development Finance Department (2016). Anchor Borrowers’ Programme Guidelines. December, 2016.

Emovwodo, S.O. (2019). The Role of Radio Programs in Agricultural Development Schemes in Osun State, Nigeria. *Journal Spektrum Komunikasi,* 7(2):1-14

FAO, IFAD, UNICEF, WFP and WHO (2021). The State of Food Security and Nutrition in the World 2021. Transforming food systems for food security, improved nutrition and affordable and healthy diets for all. Rome, FAO. DOI: <https://doi.org/10.4060/cb447en>

Kuku-Shittu, O., Mathiassen, A., Wadhwa, A., Myles, L. and Ajibola, A. (2013). Comprehensive food security and vulnerability analysis: Nigeria. IFPRI Discusion Paper 01275. Washington, DC: IFPRI.

Liverpool-Tasie, L.S.O., Omonona, B.T., Sanou, A. and Ogunleye, W.O. (2017). Is increasing inorganic fertilizer use for maize production in SSA a profitable proposition? Evidence from Nigeria. *Food Policy,* 67:41-45

Minot, N. (2002). Contract Farming in Sub-Saharan Africa: Opportunities and Challenges. Available at: <http://tinyurl.com/z5dtt8h>

Netherland Agency Enterprise (2020). Poultry Sector Study Nigeria. *Sustainable Agricultural Innovative International,* 1-50

Odunlami, H., Okuneye, P.A., Shittu, M.A., Sanusi, A.R., Elegbede, I.O. and Kies, F. (2016). Assessment of Gender Differentials in Economic and Technical Efficiency of Poultry Egg, a Case Study in Lagos State, Nigeria. *Sustainability, Agri, Food and Environmental Research,* 4(1):50-60

Ogunlela, Y.I. and Mukhtar, A.A. (2009). Gender issues in agriculture and rural development in Nigeria: The role of women. *Humanity and Social Sciences Journal,* 4(1):19-30

Olounlade, O.A., Li, G.C., Kokoye, S.E.H., Dossouhoui, F.V., Akpa, K.A.A, Anshiso, D. and Biaou, G. (2020). Impact of participation in contract farming on smallholder farmers’ income and food security in rural Benin: PSM and LATE parameter combined. *Journal of Economics and Sustainable Development*, 12(3):901-902.

Sanusi W.A (2017). The Effect of Anchor Borrower on Poverty Reduction of Smallholder Rice farmers in Lagos and Ogun State, Nigeria. *The International Journal of Organic Agriculture Research and Development,* 14:31-41.

Vaarst, M., Steenfeldt, S. and Horsted, K. (2015). Sustainable development perspectives of poultry production. *World’s Poultry Science Journal*, 71(4): 609:620.

Von Grebmer, K., Bernstein, J., Delgado, C., Smith, D., Wiemers, M., Schiffer, T., Hanano, A., Towey, O., Ni Cheilleachair, R., Foley, C., Gitter, S., Ekstrom, K. and Fritschel, H. (2021). 2021 Global Hunger Index: Hunger and Food Systems in Conflict Settings. Bonn: Welthungerhilfe; and Dublin: Concern Worldwide.

World Food Summit (1996). World Food Summit Plan of Action, paragraph 32 (g). In: Rome on World Food Security and World Food Summit Plan of Action; World Food Summit, 13-17 November 1996, Rome, Italy. Rome, FAO, 43.

Your jig saw puzzle. Osun state map jigsaw puzzle. Retrieved on October 14, 2022. [www.yourjigsawpuzzle.com](http://www.yourjigsawpuzzle.com)
