# Supplementary material for: Improving food security of farming households in Nigeria: Does broiler outgrowers’ program make any difference?
Source: PLoS One. 2023 Sep 21;18(9):e0291611. doi: 10.1371/journal.pone.0291611 (PMC10513292; doi:10.1371/journal.pone.0291611)
Supplement: Attachment — Submitted filename: correction to reviewers comments.docx [file pone.0291611.s004.docx]

Response to Reviewers’ comments

Editorial manager

| S/N | Issue raised | Correction made | Rebuttal |
| --- | --- | --- | --- |
| 1 | The entire manuscript is too lengthy and requires remarkable reduction please. The manuscript needs to be revised for typographical errors, please. The manuscript also needs to be presented using the PLOS ONE's style requirements please. The Results section needs to be separated from the Discussion section, please. Sub-headings should be deleted from the Discussion section. Please provide interpretation of your study results and possible explanations for your findings while comparing your study with results of other published works. | The needed adjustment have been made.  the entire manuscript has been modified and reorganized.  We have been able to reduce the manuscript length from 26 pages to 20 pages including the title page and abstract.  The journal format was adhere to and the entire manuscript has been proofread by a native English speaker in Canada.  She is a colleague named Jenifer Erhunmwunse. A nurse Practitioner at Micheal Garron Hospital, Toronto East Health Network, ON. E-mail: jennifercourage10@gmail.com; rhnj002@humblermail.ca. |  |
| 2` | In your Methods section, please provide additional location information, including a much clearer map drawn using geographical coordinates as opposed to copying and pasting a map from the internet (this is unacceptable please). | The map in Figure 1 has been deleted completely. In a bid to reduce the manuscript length, new map was not included in the study. |  |
| 3 | Please kindly provide evidence of ethical approval that you obtained for this study from a competent and recognized institutional review board especially since human subjects were involved in this study. Kindly provide the name of the ethical committee/institutional review board as well as approval number in the revised manuscript, please. | A sub-section of the methodology explains how ethical consent was obtained for the study. The other approval to sample beneficiaries of the programme was given by the Central bank of Nigeria. Unfortunately, the authors could not access a copy of the letter as at this time of writing because we never envisage that it will be necessary to present it in the future. Meanwhile, a copy of the consent form for the respondent was uploaded as additional file. | In my institution, only health related studies requires ethical approval from the institution authority. In agricultural economics, field survey involving farming households requires getting the consent of community leaders and trade union which we sought in this study. The community consent was obtained via a visit to the poultry farmers’ association in the commodity of study. A further survey right was obtained from the central bank of Nigeria that released the list of beneficiaries. |
| 4 | Please highlight the limitations of this study in the concluding part of the Discussion section. Also summarize the recommendations provided in the Conclusion section, please. | The limitation of the study has been fully highlighted at the concluding end of discussion section.  The commendation were also summarised separately from conclusion and highlighted in yellow pink in the revised manuscript |  |
| 5 | All the formulars and equations provided in the body of the manuscript should be deleted from the main manuscript and submitted as Supporting Information. | Equation have been submitted as supporting information and attacked to the manuscript after reference section. |  |
| 6 | Please include copies of the survey questions or questionnaires used in the study, in both the original language and English, as Supporting Information, or include a citation if they have been published previously. | A copy of the survey questionnaire have been included as an additional attachment. |  |

Reviewer 1

| S/N | Comment | Correction | Rebuttal |
| --- | --- | --- | --- |
| 1 | Table 1: Socioeconomic characteristics of smaller poultry households has part of the table at the end of one page and another part at the beginning of the next page | We have adjusted and reconciled table arrangement. It must be noted that the journal format expects every table to follow the first paragraph after mentioning. This is the reason one or two table still have to appear at border line. In addition, it was necessary to reduce the manuscript length, as such, author have to optimize the use of space. |  |
| 2 | full meaning of acronym DAYNTEE and GS FARM not stated | DAYNTEE and GS FARM are broiler processing firms’ names. We have written them in sentence case. (Dayntee Farm and GS Farm) |  |
| 3 | Table 4 has title on one page and body on another page. Table 5 is discussed under Food insecurity perceptions among poultry households but the table itself is presented under Coping mechanisms against food insecurity among poultry farming households. The tables should be properly arranged and presented. | Table arrangement and numbering have been addressed |  |
| 4 | Conclusion and recommendation stated on one page and the body on another. References stated on one page and the actual references on another page Not all intext citations were listed on the Reference page. | The separation of title and content has been addressed.  All in-text citations have been referenced. |  |

Reviewer 2

| S/N | Comment | Correction | Rebuttal |
| --- | --- | --- | --- |
| 1 | Did you used a pre-validated questionnaire for data collection or you made your own? If you made your own, have you tested it before using it for data collection? - | Diligent efforts have been made to effect every correction raised by the reviewers research instrument design and validation. The content validity was by adapting the questionnaire to the United Nation’s Food Frequency questionnaires (FFQ), while the internal validity of the instrument was done by testing for internal consistency (Cronbach alpha coefficient) |  |
| 2 | Which software (and Version) have you used for data analysis? how did test the validity of your results? | Stata package version 15, already specified in the manuscript. |  |
| 3 | Please review the conclusion and make sure that it support your findings | The conclusion and recommendation have been rewritten to reflect the findings from the study. |  |
| 4 | Since you collected data from human subject, can you provide more details about the ethical approbation? | A sub-section of the methodology explains how ethical consent was obtained for the study |  |

Reviewer 3

| S/N | Comment | Correction | Rebuttal |
| --- | --- | --- | --- |
| 1 | here was a lack of understanding and information on the "Outgrowers program" and the significance of its impact on food security. | More information has been provided on outgrowers and their significance to food security. Please, kindly check page 3, Paragraph 1 for details |  |
| 2 | No information regarding the statistics program and performed analysis. | The statistical approach and analysis were duly provided. Kindly check page 6 and 7 for details. |  |
| 3 | Abbreviations cited in the abstract were not specified or written out for clarity earlier in the abstract. | All abbreviation cited in abstract have been addressed. |  |
| 4 | The entire abstract was uninformative and failed to provide a strong understanding of what the researchers intended to achieve, how it was carried out, and how the data were created, in addition to drawing conclusions. | Thank you. The entire abstract have been rewritten and all concerned addressed |  |
| 5 | The entire document has several typographical and grammatical mistakes. | Thank you for the observation. The entire manuscript has been thoroughly formatted and proofread by a native English speaker. |  |
| 6 | - There were too many keywords. | The keywords have been reduced to 7, however, this journal permit up to 50 key words. |  |
| 7 | The entire introduction requires significant modification and reorganization. | We appreciate this. Note that the entire manuscript has been reworked. |  |
| 8 | he study's justification is unclear because it was indicated elsewhere in the report that other researchers have previously worked on the same program. | We appreciate this concern. We have adjust the justification section and make it clearer for understanding. |  |
| 9 | Additionally, it contains numerous typographical and grammatical errors | We appreciate this. Note that the entire manuscript has been reworked. |  |
| 10 | Due to the fact that human subjects were interviewed while collecting data, the materials and procedures have a significant need for ethical approval. | Thank you for your obsservation. It must be noted that all concerns about ethical issues have been addressed under a sub-section called “ethical consent for the study” | In my institution, only health related studies requires ethical approval from the institution authority. In agricultural economics, field survey involving farming households requires getting the consent of community leaders and trade union which we sought in this study. The community consent was obtained via a visit to the poultry farmers’ association in the commodity of study. A further survey right was obtained from the central bank of Nigeria that released the list of beneficiaries. |
| 11 | The tools and techniques used for data analysis are also unclear. | Thank you for the observation. The analytical tools for the study were specified in clear terms. We reported that descriptive, Food security index, and Heckman's selection model will be used for the study. Note that the food security index was meant to estimate household food security status which stood as dependent variable for Heckman's model. Secondly, Heckman's model is a 2-in-1 model. It addresses both the determinant of participation (Probit) and outcome (OLS regression) but also take into consideration the selectivity bias in the sample |  |
| 12 | Results were not presented logically for reader's for comprehension. | Thank you for your comment. The result is separated form the discussion as such, the report has become clear for layman understanding. |  |
| 13 | The entire document needs to be completely redone and formatted as a manuscript. | This has been done |  |
| 14 | It should also be subjected for a review by native English speaker. | We appreciate this comment. Note that the entire manuscript has been proofread by a native English speaker in Canada.  Name: Jenifer Erhunmwunse. A nurse Practitioner at Micheal Garron Hospital, Toronto East Health Network, ON. E-mail: jennifercourage10@gmail.com; rhnj002@humblermail.ca. |  |
