# Supplementary material for: Improving food security of farming households in Nigeria: Does broiler outgrowers’ program make any difference?
Source: PLoS One. 2023 Sep 21;18(9):e0291611. doi: 10.1371/journal.pone.0291611 (PMC10513292; doi:10.1371/journal.pone.0291611)
Supplement: Attachment — Submitted filename: Response to Reviewers111.docx [file pone.0291611.s006.docx]

**Response to Reviewers**

1. This study involves human subjects and as such an approval number/ ethics approval must be obtained from a recognized IRB as a mandatory requirement for publication. I acknowledge that you stated you have a pending submission for approval, but this manuscript publication needs that approval.

Yes. A copy of the Ethical clearance is hereby attacked to this documents.

The Ethical clearance number is IPH/OAU/12/2191. It should be note that the ethical clearance was processed after this request. Thus, it took some time to obtain the clearance.

2. If authors are stating that instrument's internal validity was tested in Ile-Ife, a city within Osun State Nigeria. Were data for this study collected from this location as well?

Thank you for your observation, The data for the study were collected from local government area in Ife community different from area where the data for validity were sampled (I.e. Modakeke area office area).

3. The style of citation used is not consistent and does not allow for ease of flow or verification and evaluation of the stated references. Please, choose a citation style and be consistent in using your preferred citation style.

All in-text citations are now given in number reference style.

4. The data set available through the link you provided is not explicit enough. It doesn't show any summary statistics or appropriate data points.

We appreciate this observation. It should be noted that all data used for this study are now included in the datasets. The summary statistics are also added as requested. Here is the link:[https://data.mendeley.com/datasets/ffffhxnjmc/1](https://data.mendeley.com/datasets/ffffhxnjmc/1). DOI:10.17632/ffffhxnjmc.1

5. Please adjust the title either on the online submission form or in your title page so that they are identical.

Thank you for your comment. The title of the papar has been adjusted both online and on the title page for uniformity. The title of the paper now reads “Improving food security of farming households in Nigeria: Does Broiler Outgrowers’ Programme makes any difference?”
