# Supplementary material for: Improving food security of farming households in Nigeria: Does broiler outgrowers’ program make any difference?
Source: PLoS One. 2023 Sep 21;18(9):e0291611. doi: 10.1371/journal.pone.0291611 (PMC10513292; doi:10.1371/journal.pone.0291611)
Supplement: Attachment — Submitted filename: response to reviewer july 7.docx [file pone.0291611.s007.docx]

Journal Requirements:

Please review your reference list to ensure that it is complete and correct. If you have cited papers that have been retracted, please include the rationale for doing so in the manuscript text, or remove these references and replace them with relevant current references. Any changes to the reference list should be mentioned in the rebuttal letter that accompanies your revised manuscript. If you need to cite a retracted article, indicate the article’s retracted status in the References list and also include a citation and full reference for the retraction notice.

Thanks for your comments. All cited papers that have been retracted were done for the following reasons.

Global Hunger Index 2021 was changed to Von Grebmer et al., 2021 who happened to be the actual authors

Liverpool-Tasie et al., 2017 was deleted due to unavailability of reference details

There was a correction involving replacing Bijman (2016) with CBN (2016) which was the original policy document for ABOP initiative in Nigeria. The error was rectified.

Consult E, and Ani NM (2017) was used to replace Ani (2017) due to omission of the first author.

Yamane, T. Statistics 1967 was added for the sampling size determination (in-text citation and listed reference)

A couple of in-text citations were moved from the result section to the discussion section after being asked to separate the two sections.

Authors such as Adepoju AO, Adejare KA (2013); Abu and Soom (2016); Emovwodo, 2019; Sanusi (2017) and Abdulazeez et al. (2018); Bature et al. (2013) and Olounlade et al. (2021) were moved.

A thorough proofreading of the manuscript was also done to minimize possible grammatical errors. All changes were highlighted in the manuscript.

Delete "well" preceding structured questionnaire. The document can either be structured, semi-structured or open-ended.

The word “well” has been deleted

Indicate or qualify the type of questionnaire utilized for data collection as stated in the abstract.

The questionnaire was qualified as structured

Clarify the number of out-growers and non-out-growers sampled for this study clearly as there seems to be a discrepancy between statements made in the 2nd and 3rd paragraphs of the material and methods.

Thank you for your observation. The number of outgrowers and non-outgrowers in the 2^nd^ paragraph of the materials and methods was well specified. On the other hand, 10 respondents each in the third paragraph addressed instrument validity only. As stated in the paper, it should be noted that these respondents were not part of the sample process for this study. In other words, they were not included in the sample size.

Clarify and explain the multistage sampling technique referred to in the methods section. For example, obtaining a list from CBN and Osun State Ministry of Agriculture cannot be considered a stage but purposely choosing all broiler farmers from Osun State can be a stage because a defined population was selected in this stage. Likewise, purposely selecting six LGAs from the Stage can also be considered a stage since a defined population was selected. Clearly share what population was selected at each stage showing exactly how the sampled population was selected.

Thank you for your comments. The multistage technique has been clearly spelt out step by step, indicating the sample techniques and populations of interest at each stage.

Delete "It is interesting to note" preceding the proportion of males heading each household in each study group sampled.

The phrase “It is interesting to note” was deleted.

Provide a credible citation for the conversion rate utilized for the Dollar to Naira rate stated in the result section.

An official conversation rate OANDA has been quoted both in-text citation and listed in the reference
