# Supplementary material for: Improving food security of farming households in Nigeria: Does broiler outgrowers’ program make any difference?
Source: PLoS One. 2023 Sep 21;18(9):e0291611. doi: 10.1371/journal.pone.0291611 (PMC10513292; doi:10.1371/journal.pone.0291611)
Supplement: Attachment — Submitted filename: Reviewers comments.docx [file pone.0291611.s008.docx]

**Reviewers’ comment/Rebuttal**

1. Please clarify the statement under the subtitle 'Food security of the outgrowers and Non outgrowers households". The outgrowers food security line was N11,098.59 ($11.24) while the non outgrowers food security line was N10,188.97 ($210.31). I assume that the statement concerning the dollar value is an error.

Thank you for your observation. The error has been rectified. The correct figure is ($10.31)

2. I acknowledge that you used a structured questionnaire to gather primary data for this study. However, you did not state the reason for your selection of the 6 LGA's for the second stage of the multi-stage sampling technique.

I aprreciate your comment. The reason for the selection of 6 LGAs was cearly stated but now modified for better understanding. It was noted that the LGs were chosen “because of the concentration of broiler growers in the area”.

3. The Reference list needs to be properly structured. Please choose a referencing style and stick to it.

Thank you very much. We have followed the reference style of the journal for 2023 publication period. The reference list has now become properly structured.

4. Please consult with the editor to determine the acceptable English style for PLOS ONE. The entire manuscript is written in a combination of American and British English styles. Similarly, the word "Study" should not be capitalized in the first line of the Materials and Methods section.

Thank you for your observation.The word “study” has been corrected to small letter. From further search, PLOS one accept American English. Therefore, the entire manuscript has been re-written in American English and every grammatical error and punctuation mark has been corrected. All changed or added words are highlighted in coloured ink.
