# Supplementary material for: Improving food security of farming households in Nigeria: Does broiler outgrowers’ program make any difference?
Source: PLoS One. 2023 Sep 21;18(9):e0291611. doi: 10.1371/journal.pone.0291611 (PMC10513292; doi:10.1371/journal.pone.0291611)
Supplement: Attachment — Submitted filename: Reviewer comment 2 Feb 2023.docx [file pone.0291611.s005.docx]

**Reviewer’s comments**

1. Despite the use of appropriate controls and sample sizes. The experiment does not seem to have appropriate replication. Sentence review needed.

Thank you. We have included multistage sampling technique used for sampling in the abstract.

2. While the limitations of this study was explained. The author did not give any suggestions that can help in overcoming these limitations in future studies.

Thank you for your observation. We have made suggestions on how we overcame the limitations and how to overcome them in the future studies.

3. Is the the poultry farmers’ association in Osun State an approved Institutional Review Board? Do they have the mandate to give ethical approbation

Actually, poultry association is not an ethical approbation authority, however, in as much as broiler anchor borrowers’ programme was practice by the members of the associations, it was necessary to obtain consent of the association in eliciting information about their operation.
Again as your study involve human participants, Have you been able to receive an official IRB approval from an established and recognized IRB? It is important that you clarify this portion of your manuscript

It must be noted that this study was carried out based on the consent obtained from the trade association and Central Bank of Nigeria however, due to the mandatory request for ethical clearance from the ethical issuing authority, we have submitted application for ethical clearance at the Institute of Public Health, College of Health Sciences, Obafemi Awolowo University, Ile-Ife, Nigeria. This process takes about 2 months for approval.

A copy of the documents submitted is hereby attached to this document as evidence.

We have decided to return our revised manuscript because the deadline given for the return of manuscript is 6^th^ of March, 2022 meanwhile, the ongoing electoral process in the country had made the university system to be on break for 3 weeks, which means our application cannot be considered for approval until mid of May, 2023. Notwitstanding, if the evidence provided is not sufficient, we implore the publishing house to give us additional 2 months to process the ethical document for approval.

4. Can you please provide the link for data access? I can't see the link to your study data, if the data are not in an repository can you share it as excel or any acceptable data management tools?

[https://data.mendeley.com/datasets/ffffhxnjmc/1](https://data.mendeley.com/datasets/ffffhxnjmc/1). DOI:10.17632/ffffhxnjmc.1

5. When citing studies in prose, could the authors consider stating the first author's name and year of publication and not and not just a reference number in the citation? An example was seen in the first paragraph of the introduction section, line 3, where the authors stated, " According to (2)."

Thank you, all citation in pros have been changed to the numbering referencing style

6. Authors should make an effort to note the locations where the instrument's internal validity was tested. Was it in a similar environment to what was intended?

Thank you for your observation. The location is Ile-Ife, a section of the study area. This has been indicated in the methodology.
